# Regulatory Effect of Iguratimod on the Balance of Th Subsets and Inhibition of Inflammatory Cytokines in Patients with Rheumatoid Arthritis

**DOI:** 10.1155/2015/356040

**Published:** 2015-12-02

**Authors:** Yunzhi Xu, Qi Zhu, Jinglve Song, Hongli Liu, Yutong Miao, Fan Yang, Feiyan Wang, Wenjing Cheng, Yebin Xi, Xiaoyin Niu, Dongyi He, Guangjie Chen

**Affiliations:** ^1^Department of Immunology and Microbiology, Shanghai JiaoTong University School of Medicine, Shanghai Institute of Immunology, Shanghai 200025, China; ^2^Guanghua Integrative Medicine Hospital, Shanghai 200052, China

## Abstract

*Objective*. To expand upon the role of iguratimod (T-614) in the treatment of rheumatoid arthritis (RA), we investigated whether the Th1, Th17, follicular helper T cells (Tfh), and regulatory T cells (Treg) imbalance could be reversed by iguratimod and the clinical implications of this reversal.* Methods*. In this trial, 74 patients were randomized into iguratimod-treated (group A) and control (broup B) group for a 24-week treatment period. In the subsequent 28 weeks, both groups were given iguratimod. Frequencies of Th1, Th17, Tfh, and Treg were quantified using flow cytometry, and serum cytokines were detected by enzyme-linked immunosorbent assay. mRNA expression of cytokines and transcriptional factor were quantified by RT-PCR. The composite Disease Activity Score, erythrocyte sedimentation rate, and C-reactive protein were assessed at each visit.* Result*. The clinical scores demonstrated effective suppression of disease after treatment with iguratimod. In addition, iguratimod downregulated Th1, Th17-type response and upregulated Treg. Furthermore, the levels of Th1, Th17, and Tfh associated inflammatory cytokines and transcription factors were reduced after treatment with iguratimod, while the levels of Treg associated cytokines and transcription factors were increased.

## 1. Introduction

Rheumatoid arthritis (RA) is a chronic autoimmune inflammatory disease, accompanied by progressive destruction of joint cartilage and bone, ultimately leading to functional impairment and disability [1, 2]. Numerous studies have demonstrated that cell-cell and cytokine networks established in the inflamed RA synovium are responsible for disease chronicity, autoimmune response, and damage to bone and cartilage [3–5]. Moreover, the abundance of T cells within the mononuclear infiltrates of the hyperplastic synovial membrane, taken together with the local production of T cell-derived cytokines, suggests that T cells are important in the autoimmune response in RA [6]. T cell lineages include Th1, Th17 cell, CD4^+^CD25^+^ regulatory T cell (Treg), and the recently reported follicular helper T cell (Tfh). While RA is characterized by a marked shift towards the Th1 phenotype, the specific contributions to RA remain incompletely characterized [7]. IL-17 is the main cytokine secreted by Th17 cells. Studies have reported higher concentration of IL-17 in the serum and synovial fluid of RA patients compared to control populations or osteoarthritis (OA) patients [8, 9]. Most studies reported that the percentage of circulating Treg in RA is reduced compared to healthy people, but some other studies observed either an increase or similar cell percentages compared to healthy controls or OA patients [10–16]. Follicular helper T cells (Tfh) present in the germinal center have been shown to contribute to B cells proliferation and affinity maturation. Tfh dysregulation is involved in the development of autoimmune pathologies, such as systemic lupus erythematosus, rheumatoid arthritis, and other autoimmune diseases [17].

Iguratimod (T-614) is a small-molecule, antirheumatic drug which acts to suppress tumor necrosis factor-alpha-induced production of inflammatory cytokines in cultured human synovial cells and human THP-1 cells [18–20]. It also reduces immunoglobulin (Ig) production by acting on B cells without affecting B cells proliferation [21]. Hara et al., previously demonstrated a 20% improvement in ACR criteria (ACR20) in iguratimod-treated Japanese patients with active rheumatoid arthritis. These results were comparable to those improvements achieved by salazosulfapyridine (63.1% for iguratimod versus 57.7% for salazosulfapyridine) [22]. Furthermore, iguratimod reduced rheumatoid factor (RF) and production of IgG, IgM, and IgA in patients with RA [22]. In addition, iguratimod has anti-inflammatory effects and improves immunological abnormalities in animal models with arthritis or autoimmune disease [23, 24]. Thus we compared the efficacy of iguratimod against a placebo treatment in Chinese patients with active rheumatoid arthritis.

## 2. Methods

### 2.1. Patients

This study conformed to the approved institutional guidelines. 74 RA patients fulfilling the 2010 diagnostic criteria of the American College of Rheumatology (ACR) were included in the study [25]. Informed consent was obtained from each patient. All the patients had active disease and were consistent with the criteria of activity: (1) ≥6 joints were swollen; (2) ≥6 joints were tender; and (3) they fit two of the three criteria: (a) on the day of visiting, morning stiffness lasting more than 45 min; (b) erythrocyte sedimentation rate (ESR) ≥28 mm/h; and (c) C-reactive protein (CRP) ≥20 mg/dL.

**Figure 1:**
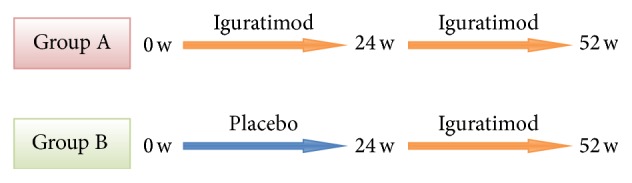
The difference of administration between groups A and B.

All 74 patients were randomized into either group A or group B. Iguratimod was administered to patients in group A and placebo was administered to patients in group B for the first 24 weeks. Iguratimod was administered to patients in both groups for the subsequent 28 weeks (Figure 1).

### 2.2. Blood Sample

All blood samples were taken in the morning of the injection day in a fasting state. The samples were collected into collection tubes containing 0.2 mL sodium heparin. Peripheral blood mononuclear cells (PBMCs) were prepared by Ficoll density gradient for flow cytometric analysis. Plasma was obtained after centrifugation and stored at −40°C for the measurement of cytokines.

### 2.3. Flow Cytometric Analysis of Th1, Th17, Tfh, and Treg

#### 2.3.1. Cell Preparation

For the analysis of Th1, Th17, Tfh, and Treg, PBMCs were suspended at a density of 1 × 10^7^ cells/mL in complete culture medium (RPMI 1640 supplemented with 100 U/mL penicillin and 100 *μ*g/mL streptomycin, 2 mM glutamine and with 10% heat-inactivated fetal calf serum, Gibco BRL).

#### 2.3.2. Surface and Intracellular Staining

For Th1 and Th17 analysis, cells were stimulated for 4-5 h with 50 ng/mL PMA (Sigma-Aldrich, St. Louis, MO, USA), 750 ng/mL ionomycin (Calbiochem, La Jolla, CA, USA), and Golgiplug at the recommended concentrations (BD Pharmingen, San Diego, CA, USA) at 37°C under a 5% CO_2_ environment. Then cells were stained with Percp cy5.5-conjugated anti-human CD4 (BD Pharmingen, San Diego, CA, USA) at room temperature for 15 min, fixed and permeabilized with Cytofix/Cytoperm solution (BD Pharmingen, San Diego, CA, USA), and then labeled with APC-conjugated anti-human IFN-*γ* (Biolegend, San Diego, CA, USA) and PE-conjugated anti-human IL-17 (Santa Cruz Biotechnology, CA, USA). For Treg analysis, cells were labeled with Percp cy5.5-conjugated anti-human CD4 and PE-conjugated anti-human CD25 (BD Pharmingen, San Diego, CA, USA). For intracellular Foxp3 staining, cells were fixed and permeabilized according the manufacturer's protocol for the staining set (eBioscience, San Diego, CA, USA) and labeled with FITC-conjugated anti-human Foxp3 (eBioscience, San Diego, CA, USA). For Tfh analysis, cells were incubated with Percp cy5.5-conjugated anti-human CD4, FITC-conjugated anti-CCR7 (BD Pharmingen, San Diego, CA, USA) and APC-conjugated anti-PD-1 (BD Pharmingen, San Diego, CA, USA). The percentage of positive stained cells was analyzed using a FACS instrument (BD Biosciences, San Jose, CA, USA).

### 2.4. Cytokine Measurement

The plasma levels of GM-CSF, IFN-*γ*, IL-12p70, IL-13, IL-1*β*, IL-2, IL-21, IL-22, IL-23, IL-27, IL-5, IL-9, IL-18, TNF-*α*, IL-4, IL-10, IL-6, and IL-17A were measured by multiplex immunoassay using kits from eBioscience, following the manufacturer's instructions. The concentrations were calculated from a standard curve according to the manufacturer's protocol.

### 2.5. RNA Isolation

Total RNA was isolated using RNeasy Mini Kit (Qiagen) from PBMCs (1 × 10^6^). Genomic DNA was removed from total RNA prior to cDNA synthesis using RNase-free DNase Set (Qiagen). First-strand cDNA synthesis was performed for each RNA sample using Sensiscript RT Kit (Qiagen). Random hexamers were used to prime cDNA synthesis.

### 2.6. Real-Time RT-PCR Analysis of Gene Expression

Primer Express software (ABI) was used to design primers from published cDNA sequences. BLASTN searches were conducted on the primer nucleotide sequences to ensure gene specificity. The primer sequences were as follows:

 GAPDH, forward 5′-AGGTCGGTGTGAACGGATTTG-3′ and reverse 5′-TGTAGACCATGTAGTTGAGGTCA-3′;
 Bcl6, forward 5′-TGAAGCAAGGCATTGGTGAA-3′ and reverse 5′-GACGATTAAGGTTGAGAAGAACATCA-3′;
 IL-21, forward 5′-GGGAGAAGACAGAAACACAGAC-3′ and reverse 5′-CCACTTGGAATACAAZGAAATG-3′;
 IL-17, forward 5′-ACCTGTGTCACCCCGATTG-3′ and reverse 5′-CCGCTTTTTCACCTTTTTCTAA-3′;
 ROR*γ*t, forward 5′-ACTTTTCCGAGGATGAGATTGC-3′ and reverse 5′-AGCGGCTTGGACCACGAT-3′;
 Foxp3, forward 5′-CGACCCCCTTTCACCTACG-3′ and reverse 5′-CCCTTCTCGCTCTCCACC-3′;
 STAT3, forward 5′-ATTGACAAAGACTCTGGGGACG-3′ and reverse 5′-CTTGGTGGTGGAGGAGAACTG-3′;
 T-bet, forward 5′-GATGAAAGGAACAGAAACAGTG-3′ and reverse 5′-GAAACCAAAAGCAAGACGC-3′.

Relative quantification of gene expression was performed using the ABI Prism 7900 sequence detection system. SYBR Green master mix (ABI) was used for real-time RT-PCR to detect the abundance of PCR products among samples. Thermocycler conditions comprised an initial holding at 50°C for 2 min and then 95°C for 10 min. This was followed by a 2-step PCR program consisting of 95°C for 15 s and 60°C for 60 s for 35 cycles. Data were collected and quantitatively analyzed on an ABI Prism 7900 HT sequence detection system (ABI). The GAPDH gene was used as an endogenous control to normalize the differences in the amount of total RNA in each sample. All quantities were expressed as *n*-fold relative to a calibrator.

### 2.7. Clinical Assessments

Laboratory measurements, including ESR and CRP, were assessed at each visit. Disease activity was assessed by the composite 28-joint count Disease Activity Score (DAS28) and was measured prior to the iguratimod treatment.

### 2.8. Statistical Analysis

Student's *t*-test was used to analyze the differences between groups. One-way ANOVA was initially performed to determine whether an overall statistically significant change existed before using the two-tailed paired or unpaired Student's *t*-test. A value of *p* < 0.05 was considered statistically significant.

## 3. Results

### 3.1. Disease Characteristics of RA Patients

A total of 30 patients in group A and 27 patients in group B completed their participation in the trial. In group A, 7 patients did not complete their enrollment trial (one patient withdrew due to informed consent, 2 patients had no effect, 2 patients had increased level of ALT, 1 patient had decreased level of white blood cells, and 1 patient had decreased level of platelets). In group B, 10 patients quit the trial (one patient withdrew informed consent, 7 patients had no effect, 1 patient had increased level of ALT, and 1 patient had pulmonary infection).

There are 18 women in group A and 17 women in group B. The average age of groups A and B is 45 ± 13 and 45 ± 12, respectively.

### 3.2. Assessment of RA Patients Undergoing Iguratimod Treatment

The assessment of the patients is summarized in Tables 1 and 2.

### 3.3. Frequencies of CD4^+^ T Cell Subsets in Peripheral Blood of Patients before and after Therapy

**Figure 2:**
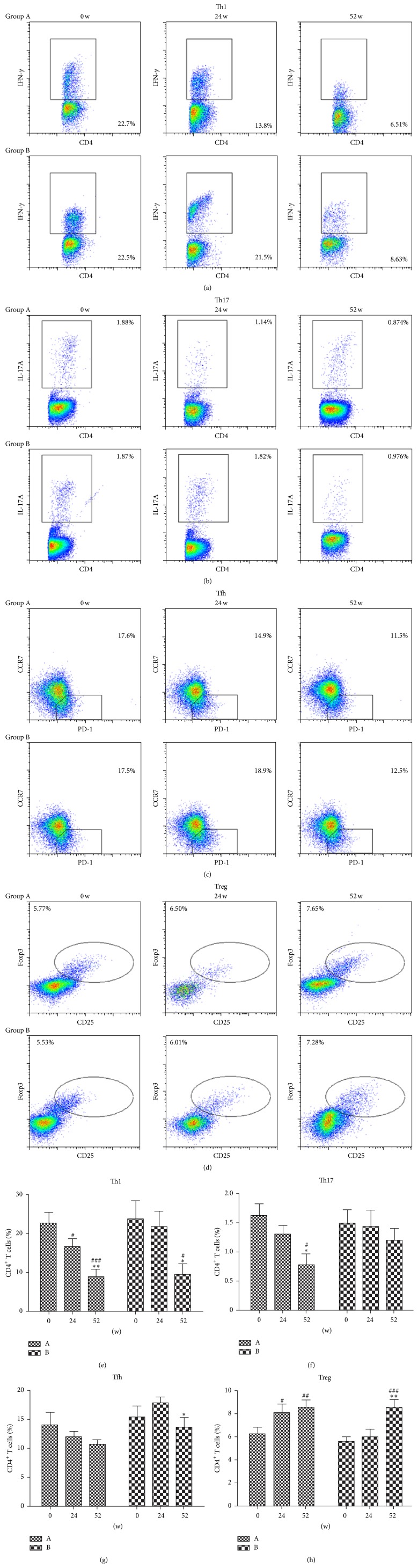
Frequencies of CD4^+^T cell subset in RA patients before and after therapy. The proportion of IFN-*γ* ^+^Th1, IL-17^+^Th17, circulating CD4^+^CCR7^low^PD-1^high^Tfh, and CD4^+^CD25^+^Foxp3^+^Treg in CD4^+^T cells was analyzed by flow cytometry on 0 w, 24 w, and 52 w. (a) Representative staining data showing the proportion of Th1. (b) Representative staining data showing the proportion of Th17. (c) Representative staining data showing the proportion of Tfh. (d) Representative staining data showing the proportion of Treg. (e) Collated data showing the proportion of Th1. (f) Collated data showing the proportion of Th17. (g) Collated data showing the proportion of Tfh. (h) Collated data showing the proportion of Treg. ^*∗*^ *p* < 0.05, ^*∗∗*^ *p* < 0.01, and ^*∗∗∗*^ *p* < 0.001 are statistically significant compared to 24 w. ^#^ *p* < 0.05, ^##^ *p* < 0.01, and ^###^ *p* < 0.001 are statistically significant compared to 0 w. Group A: iguratimod was administered to patients for 52 weeks. Group B: placebo was administered to patients for the first 24 weeks and iguratimod was administered to patients for the subsequent 28 weeks.

As shown in Figure 2, the percentages of IFN-*γ* ^+^Th1 cells, IL-17^+^Th17 cells, and circulating CD4^+^CCR7^low^PD-1^high^Tfh among CD4^+^T cells were lower in peripheral blood of RA patients after 52 weeks of therapy with iguratimod in group A. This effect was most pronounced for IFN-*γ* ^+^Th1 cells. The percentages of CD4^+^CD25^+^Foxp3^+^Treg among CD4^+^T cell subsets were significantly higher in peripheral blood of RA patients after the treatment than those in RA patients before the treatment in group A. The percentages of IFN-*γ* ^+^Th1 cells, IL-17^+^Th17 cells, and circulating CD4^+^CCR7^low^PD-1^high^Tfh among CD4^+^T cells were no significantly changed in peripheral blood of RA patients after the first 24-week therapy with placebo in group B. However, the percentages of IFN-*γ* ^+^Th1 cells were significantly decreased and the percentages of CD4^+^CD25^+^Foxp3^+^Treg were significantly increased in peripheral blood of RA patients after the following 28-week therapy with iguratimod in group B.

### 3.4. Changes of Cytokines in Serum of Patients before and after Therapy

**Figure 3:**
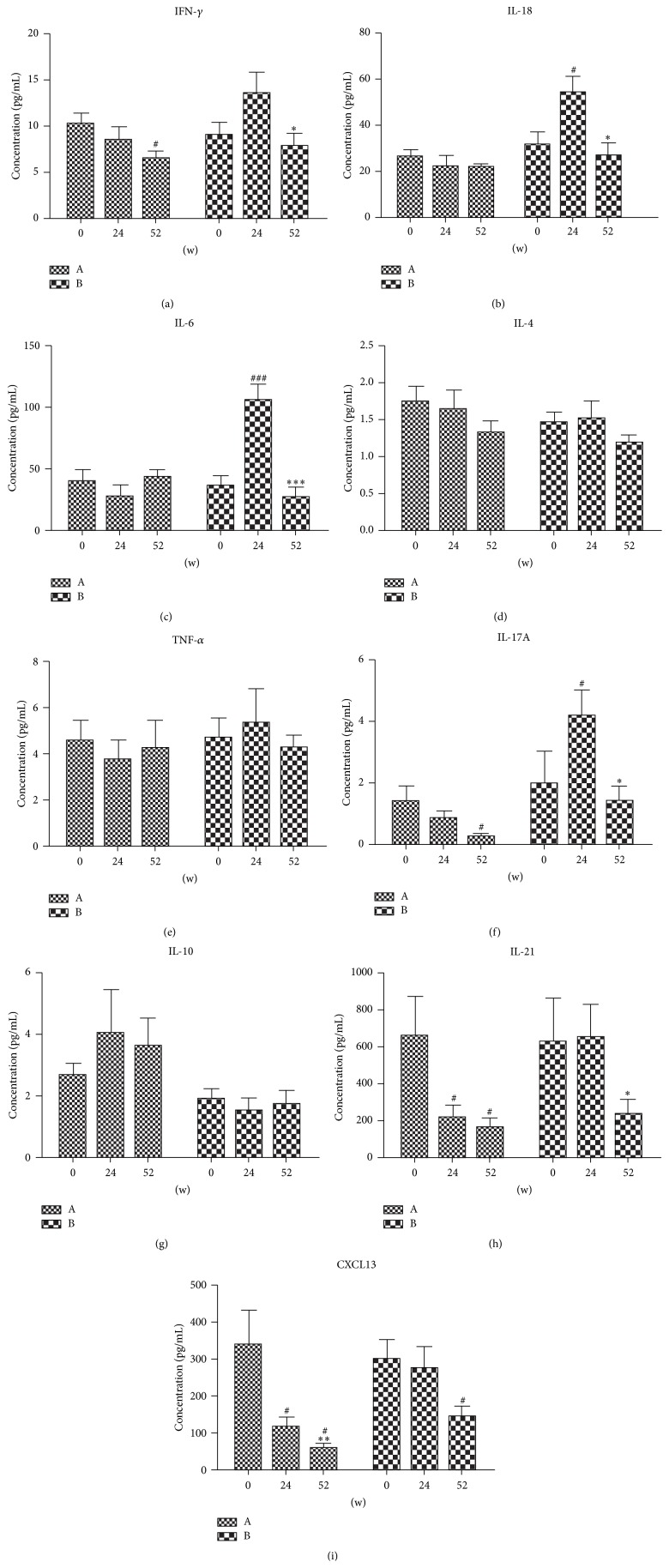
Changes of cytokines in serum of RA patients before and after therapy. Concentration (pg/mL) of (a) IFN-*γ*, (b) IL-18, (c) IL-6, (d) IL-4, (e) TNF-*α*, (f) IL-17A, (g) IL-10, (h) IL-21, and (i) CXCL13. Mean concentrations of cytokines in serum of patients in our study were quantified by ELISA, and ^*∗*^ *p* < 0.05, ^*∗∗*^ *p* < 0.01, and ^*∗∗∗*^ *p* < 0.001 are statistically significant compared to 24 w. ^#^ *p* < 0.05, ^##^ *p* < 0.01, and ^###^ *p* < 0.001 are statistically significant compared to 0 w. Group A: iguratimod was administered to patients for 52 weeks. Group B: placebo was administered to patients for the first 24 weeks and iguratimod was administered to patients for the subsequent 28 weeks.

Eighteen cytokines were evaluated prior to and following therapy. We were unable to detect GM-CSF, IL-12p70, IL-13, IL-1*β*, IL-2, IL-22, IL-23, IL-27, IL-5, and IL-9 due to low concentration. Serum cytokines were analyzed in both RA patients of group A and group B. As shown in Figure 3, the serum levels of proinflammatory cytokines such as IFN-*γ* (Figure 3(a)), IL-18 (Figure 3(b)), IL-6 (Figure 3(c)), IL-17A (Figure 3(f)), and IL-21 (Figure 3(h)) were degraded after the therapy in group A and the concentrations of IFN-*γ* (Figure 3(a)), IL-17A (Figure 3(f)), and IL-21 (Figure 3(h)) were significantly reduced after 52-week therapy. In group B, serum levels of proinflammatory cytokines such as IFN-*γ* (Figure 3(a)), IL-18 (Figure 3(b)), IL-6 (Figure 3(c)), IL-17A (Figure 3(f)), and IL-21 (Figure 3(h)) were degraded significantly after the 28-week therapy while the concentrations of IFN-*γ* (Figure 3(a)), IL-18 (Figure 3(b)), IL-6 (Figure 3(c)), and IL-17A (Figure 3(f)) were increased after first 24-week therapy with placebo, especially IL-18 (Figure 3(b)) and IL-6 (Figure 3(c)). No significant changes were observed in the serum levels of TNF-*α* (Figure 3(e)) and anti-inflammatory cytokines such as IL-4 (Figure 3(d)) and IL-10 (Figure 3(g)) in both groups A and B.

The chemokine C-X-C motif chemokine 13 (CXCL13) is essential for follicle formation [26]. Recently, CXCL13 has risen to be a possible novel marker of disease and inflammation in RA. CXCL13 was found upregulated in RA patients and was suggested to be related with both disease activity and rheumatoid factors (RF) [27, 28]. As shown in Figure 3(i), the concentration of CXCL13 was greatly reduced after 52-week therapy in group A. In group B, serum level of CXCL13 was significantly reduced after the 28 weeks while it has no change after first 24-week therapy.

### 3.5. Changes of the Expression of Th1, Th17, Tfh, Treg, Related Transcriptional Factors, and Cytokines in Patients before and after Therapy

**Figure 4:**
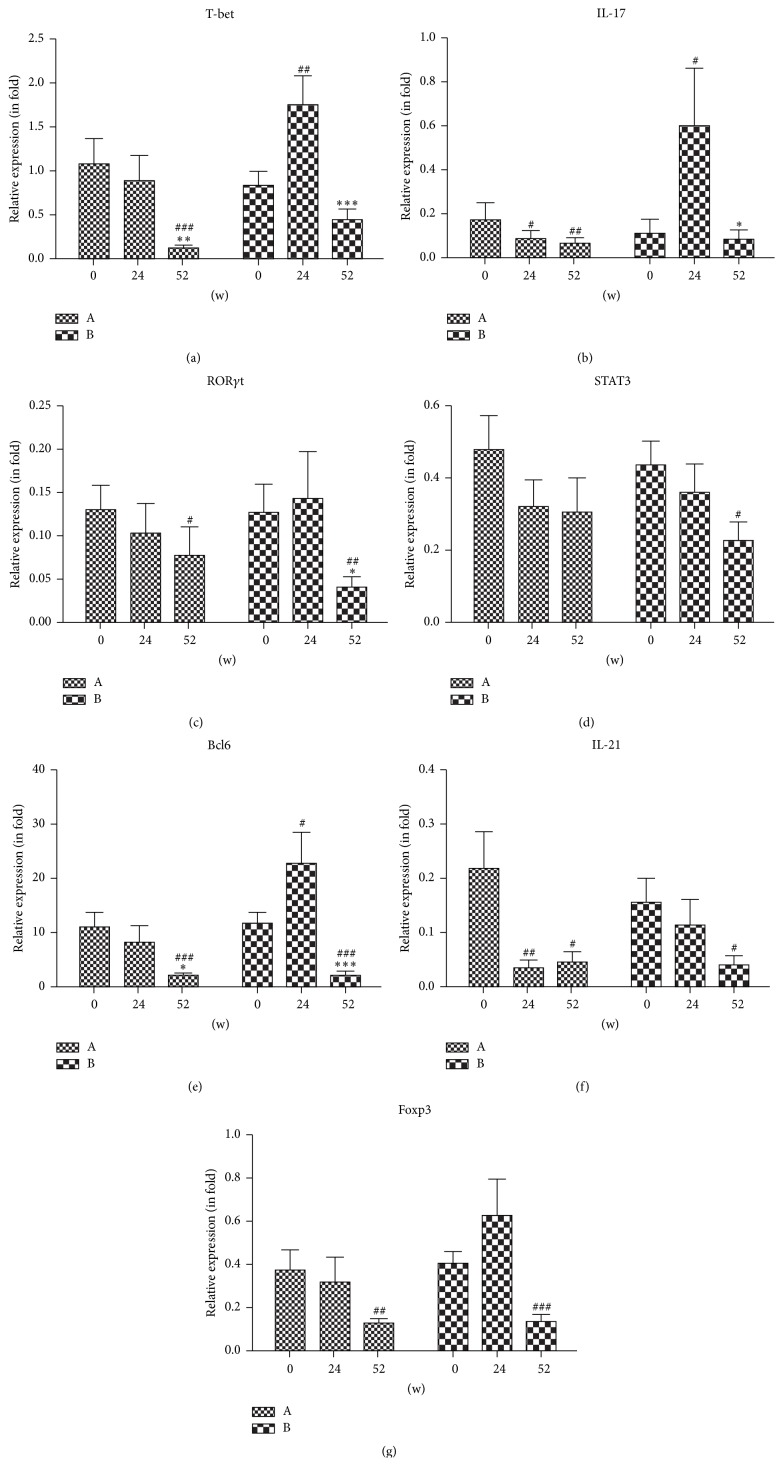
Changes of the expression of Th1/Th17/Tfh/Treg related transcriptional factors at mRNA levels in RA patients before and after therapy. Relative expression of (a) T-bet, (b) IL-17, (c) ROR*γ*t, (d) STAT3, (e) Bcl6, (f) IL-21, and (g) Foxp3. Mean mRNA expressions of cytokines and transcription factors in our study were quantified by RT-PCR and are statistically significant compared to 24 w (^*∗*^ *p* < 0.05, ^*∗∗*^ *p* < 0.01, or ^*∗∗∗*^ *p* < 0.001). ^#^ *p* < 0.05, ^##^ *p* < 0.01, and ^###^ *p* < 0.001 are statistically significant compared to 0 w. Group A: iguratimod was administered to patients for 52 weeks. Group B: placebo was administered to patients for the first 24 weeks and iguratimod was administered to patients for the subsequent 28 weeks.

In an effort to characterize the biological changes induced by therapy we evaluated the change in expression of Th1, Th17, Tfh, Treg related transcriptional factors, and cytokines in RA patients before and after therapy. As shown in Figure 4, the mRNA expression of T-bet (Figure 4(a)), IL-17 (Figure 4(b)), ROR*γ*t (Figure 4(c)), STAT3 (Figure 4(d)), Bcl6 (Figure 4(e)), and IL-21 (Figure 4(f)) decreased significantly in iguratimod-treated patients compared with those in patients without treatment or treated with placebo. Interestingly, iguratimod also reduced the expression of Treg related transcriptional factors Foxp3 (Figure 4(g)).

## 4. Discussion

Rheumatoid arthritis is a systemic inflammatory disease, presumably of autoimmune origin. Iguratimod is a novel disease-modifying antirheumatic drug, which has been used for the treatment of rheumatoid arthritis exclusively in Japan and China [29]. Numerous studies have demonstrated that both the Disease Activity Score (DAS) 28-erythrocyte sedimentation rate and DAS28-C-reactive protein (CRP) are significantly decreased after iguratimod treatment [30, 31]. Our data is in agreement with previous reports. Our results show that ACR20, ACR50, and ACR70 in the iguratimod-treated group (group A) were improved more than those of group B at 24 weeks. The patients in group B were also given iguratimod in the last 28 weeks.  Table 1 highlights the improvement of ACR20, ACR50, and ACR70 at week 52 as compared to those at week 24. Notably, the ACR20, ACR50, and ACR70 in group A had little changes. Iguratimod significantly decreased DAS28, ESR, CRP, RF, and the production of IgG, IgM, and IgA at previous 24 weeks. But the ESR, CRP, and the concentration of RF, IgM, and IgA at week 52 were increased. The DAS28 and the production of IgG at week 52 were similar to that of week 24. In group B the DAS28 and the production of IgG, IgM decreased a little while CRP, RF, and IgA increased a lot at week 24. After iguratimod treatment, all these measures were improved. These results highlight the efficacy of a 24-week extension phase with iguratimod.

The antiarthritic mechanism of iguratimod has not previously been elucidated. Our experimental assessment shows downregulation of autoantibody following iguratimod treatment, indicating the clinical value of this approach. Antibody responses can be induced in a T cell dependent or independent manner. T cells play an important role in RA [32–34]. Previous studies have found that CD4^+^ T cells, especially Th1 cells, are thought to be central to the pathogenesis of RA [35]. However, more recent studies have revealed proinflammation cytokines secreted mainly by Th1 cells were scarcely detected in the affected joints of RA [36]. Since Th17 cells were reported in 2005, much attention has been placed on understanding the characteristics and functions of Th17/IL-17 on autoimmune-like RA [37]. Recently studies have found that the level of IL-17 increased in RA patients and CIA mice [34]. Treg is essential for the maintenance of peripheral tolerance and for control of the immune response. We hypothesized that iguratimod could act on T cell subset and influence the secretion of T cell related cytokines. In this report, we present an analysis of Th1, Th17, Treg and Tfh, and their relative cytokines in RA patients receiving iguratimod therapy. We demonstrate that iguratimod therapy significantly decreases Th1, Th17 cell frequencies while significantly increasing the frequencies of Treg. We also evaluated mRNA expression of key transcription factors by real-time PCR and found that T-bet and ROR*γ*t were significantly downregulated. Interestingly, the mRNA expression of Treg related transcription factor Foxp3 was reduced. We hypothesize that the mRNA is degraded quickly but the protein is degraded at a much slower rate. Because of limited T cells, further functional validation will be necessary in subsequent investigations.

Tfh are a specialized subpopulation of CD4^+^ Th cells that localize to GCs where they secrete IL-21, a cytokine critical for GC development and the formation of memory B and plasma cells [38, 39]. There is some evidence in both mice and humans that iguratimod inhibits nuclear factor-*κ*B activity and reduces immunoglobulin production by acting directly on B cells without modifying B cell proliferation [19, 21]. Walsh et al. found that LKB1 inhibition of NF-*κ*B in B cells prevents T follicular helper cell differentiation and germinal center formation [40]. The expression changes we observed, in conjunction with the Tfh in CD4^+^ T cells, were in accordance with previous reports. Tfh frequency in group B was reduced while that of group A was not significantly affected.

Evidence shows that T cell expansion and differentiation maybe occur within the synovial membrane as a result of a favorable cytokine environment [41]. Iguratimod is a small-molecule drug that can suppress inflammatory cytokines [42, 43]. We found that preinflammation cytokines IFN-*γ*, IL-18, IL-6, and IL-17A were downregulated by iguratimod. Moreover, IL-17 and IL-21 expression was also downregulated at the mRNA level. The change of cytokine expression is consistent with the frequency of Th subsets. It has been reported that iguratimod can inhibit the production of TNF-*α*, which has been found to inhibit the suppressive function of CD4^+^CD25^+^Treg [6]. In addition, Treg exert suppressive activity on self-reactive T cells through secreting TGF-*β*, IL-10, and CTLA4-dependent negative-feedback loop [44, 45]. Our data shows no significant changes of the concentration of TNF-*α* and IL-10, but their trend is corresponding to previous studies. CXCL13 is essential for follicle formation and acts as a possible novel marker of disease and inflammation in RA. CXCL13 was found upregulated in RA patients and was suggested to be related with both disease activity and RF. The reduced concentration of CXCL13 after iguratimod treatment is the evidence of decreased ectopic lymphoid.

In conclusion, iguratimod can improve clinical and experimental assessment of RA patients. Furthermore, it can mediate immunity balance by suppressing proinflammation Th subset cells and related cytokines and upregulating Treg and anti-inflammation cytokines. However, the time iguratimod works is limited and it needs further studies.

## Figures and Tables

**Table 1:** Clinical assessment of RA patients undergoing iguratimod treatment.

	Group A			Group B		
	0 w	24 w	52 w	0 w	24 w	52 w
DAS28	6.11 ± 0.69	3.97 ± 1.31	3.85 ± 1.70	6.04 ± 0.72	5.65 ± 1.27	3.76 ± 1.69
ACR response rate, % of patients						
ACR20		70.00	80.00		48.15	77.78
ACR50		56.67	56.67		18.52	50.00
ACR70		26.67	20.00		7.41	22.22

Clinical responses were defined according to the American College of Rheumatology 20% response criteria (ACR20), ACR50, and ACR70.

**Table 2:** Experimental assessment of RA patients undergoing iguratimod treatment.

	Group A			Group B		
	0 w	24 w	52 w	0 w	24 w	52 w
ESR (mm/h)	40.48 ± 19.7	20.21 ± 12.84	28.14 ± 19.09	38.29 ± 16.89	37.63 ± 31.22	27.59 ± 27.96
CRP (mg/L)	17.98 ± 16.35	10.18 ± 13.93	17.17 ± 18.83	22.40 ± 22.93	24.38 ± 27.84	12.65 ± 18.66
RF (IU/mL)	357.79 ± 540.61	221.83 ± 461.93	248.74 ± 463.33	215.71 ± 229.22	330.92 ± 373.91	162.70 ± 254.67
IgG (g/L)	17.10 ± 4.55	14.24 ± 3.11	14.13 ± 2.93	16.11 ± 3.63	15.81 ± 3.99	14.07 ± 3.80
IgM (g/L)	1.98 ± 1.67	1.46 ± 0.99	1.51 ± 0.92	1.75 ± 0.88	1.71 ± 0.73	1.66 ± 1.35
IgA (g/L)	3.47 ± 1.11	2.82 ± 1.16	2.89 ± 1.22	2.87 ± 1.17	3.17 ± 1.68	2.17 ± 1.13

ESR: erythrocyte sedimentation rate; CRP: C-reactive protein; RF: rheumatoid factor.

## Abbreviations

RA: Rheumatoid arthritis
Treg: Regulatory T cell
Tfh: Follicular helper T cell
OA: Osteoarthritis
Ig: Immunoglobulin
RF: Rheumatoid factor
ACR: American College of Rheumatology
ESR: Erythrocyte sedimentation rate
CRP: C-reactive protein
PBMC: Peripheral blood mononuclear cell
DAS28: Composite 28-joint count Disease Activity Score.
